# Emotional exhaustion in front-line healthcare workers during the COVID-19 pandemic in Wuhan, China: the effects of time pressure, social sharing and cognitive appraisal

**DOI:** 10.1186/s12889-021-10891-w

**Published:** 2021-04-30

**Authors:** Huan Wang, Xinyao Zhou, Xiuli Jia, Caiping Song, Xu Luo, Hua Zhang, Hao Wu, Junying Ye

**Affiliations:** 1 Development and Planning Department, Chongqing Medical University, Chongqing, China; 2 School of Economics and Management, Wuhan University, Wuhan, China; 3 Neurology Department, The 987 Hospital of PLA, Baoji, China; 4 Xinqiao Hospital, Army Medical University, No. 83 Xinqiao Zhengjie, Shapingba District, Chongqing, China; 5 Department of Medical Administration, Southwest Hospital, Army Medical University, Chongqing, China; 6 Chongqing Health Center for Women and Children, No.120 Longshan Road, Yubei District, Chongqing, China

**Keywords:** COVID-19, Healthcare workers, Occupational stress, Mental fatigue, Social support, Cognition

## Abstract

**Background:**

With the increasing spread of COVID-19, healthcare workers, especially front-line medical staff, have become more vulnerable to emotional exhaustion.

**Objectives:**

This study aimed to determine the influence of time pressure on the emotional exhaustion of front-line healthcare workers, and explore the effects of social sharing and cognitive reappraisal on this.

**Methods:**

This cross-sectional study was conducted in March 2020. A total of 232 questionnaires were completed by front-line healthcare workers in Wuhan city, Hubei province, China. Hierarchical linear regression and conditional process analysis were performed to explore the relationships among time pressure, social sharing, cognitive reappraisal, and emotional exhaustion.

**Results:**

Time pressure was positively associated with social sharing and emotional exhaustion. Social sharing presented the dark side, a negative effect that was always kept concealed, in terms of the impact on emotional exhaustion. Cognitive reappraisal negatively moderated the relationship between time pressure and social sharing, and it further indirectly influenced the relationship between time pressure and emotional exhaustion through social sharing.

**Conclusions:**

Our findings shed light on how time pressure influences the emotional exhaustion of healthcare workers during the COVID-19 period. Although social sharing is commonly regarded as a positive behavior, we identified a dark side in terms of its impact. We also identified that improving cognitive reappraisal may present a positive strategy toward alleviating emotional exhaustion.

## Introduction

The COVID-19 pandemic has received worldwide attention. The increased workload has resulted in greater pressure and emotional issues for healthcare workers [1, 2]. However, little theoretical or empirical work has been devoted to understanding the associations between this pressure and the emotional experiences of healthcare workers during the COVID-19 period.

Healthcare workers are vulnerable to emotional exhaustion. In Britain, between 31 and 54.3% of doctors reported experiencing high emotional exhaustion before the COVID-19 pandemic [3], and as such, exhaustion is likely to be even higher at the current time. Research has demonstrated that front-line medical staff fighting against COVID-19 experience high levels of anxiety and depression [4, 5]. Thus, emotional exhaustion among healthcare workers is associated with a variety of occupational stresses that are likely to increase during the COVID-19 pandemic [6, 7].

In China, more than 40,000 medical staff went to Hubei province to assist with COVID-19, which was regarded as one of the fastest spreading, most extensive, and most challenging public health emergencies China has ever encountered. During the fight against COVID-19, front-line healthcare workers are not only confronting increasing workload and infection risk but also struggling with the exhaustion of their emotional resources. Recently, researchers have called for more attention to be paid to the mental health of first-line medical staff [8–10]. Therefore, the current state of healthcare workers’ emotional exhaustion, and the key influencing factors and mechanisms of this pressure-emotion mechanism, need to be urgently investigated.

Having a social support network can lead to more effective coping strategies to deal with stress. People under stress can gain a feeling of attachment, care, or help received from interpersonal relationships through social support. One way to gain social support is through social sharing, which occurs when people share their experiences and feelings with others after experiencing emotional events [11], and in particular, negative events [12].

It is unclear how social sharing affects the emotional exhaustion of front-line healthcare workers specifically in the COVID-19 context. Previous studies have regarded social sharing as a positive behavior. For example, a survey of call center employees found that social sharing helped to deplete employee emotional burnout after experiencing work stressors [13]. However, social sharing was recently associated with further deterioration to employee emotional exhaustion when confronting customer mistreatment [12]. According to the conservation of resources theory [14], healthcare workers are more likely to regard time pressure as a threat associated with losing or depleting their resources, which would bring a significant psychological burden. To confront that threat, they would take action such as engaging in social sharing to defend, conserve, and acquire their valued resources. However, social sharing may have the undesired effect of prolonging the negative experience. Therefore, in consideration of front-line healthcare workers during the COVID-19 pandemic, the effect of social sharing on emotional exhaustion remains unknown.

According to emotion regulation theory [15], certain measures can be taken to alleviate the impact of negative events on emotion. Front-line healthcare workers are under heavy rescue burden as they not only put themselves at risk for COVID-19 but also have to rescue increasing patients during a very tense time. As one of the main methods of emotional regulation, cognitive reappraisal enables individuals to appraise stress from a positive perspective. Under the influence of cognitive reappraisal, front-line healthcare workers may reinterpret both their rescue burden and stress as the noble mission of saving lives, thus giving a sense of higher value to their work. As such, people would decrease in their complaining about and sharing of negative events with others as a result of engaging in cognitive reappraisal.

Medical staff, as the main force in facing the potential long-term prevalence of COVID-19 and other unknown infectious diseases [16], require greater attention to be paid to their own health. Our study aimed to explore the relationships among time pressure, emotional exhaustion, social sharing, and cognitive reappraisal, as a means to deepen our understanding of the characteristics and related mechanisms of healthcare workers’ emotional exhaustion during the COVID-19 pandemic. This work also aimed to provide a theoretical foundation for the management of healthcare workers’ emotional experience as well as the development of psychological interventions, which would be beneficial in improving the emergency response capacity of the public health system.

### Theoretical framework

**Fig. 1:**
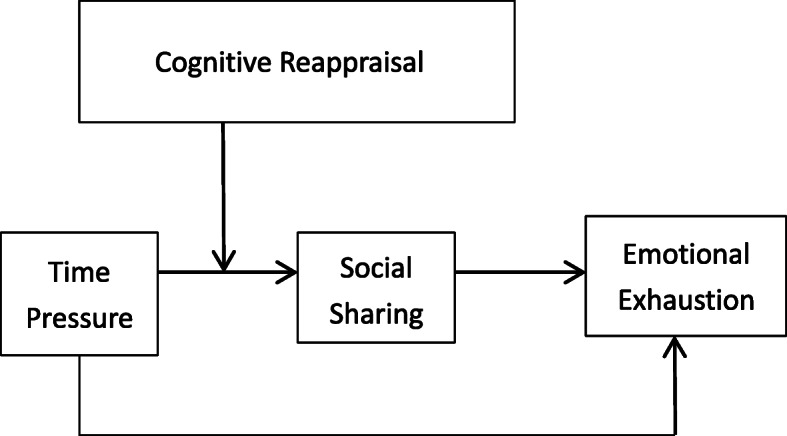
Theoretical Model

A theoretical model (see Fig. 1) was established to examine three research questions. First, we aimed to identify the influence of time pressure on emotional exhaustion and explore the potential mediating mechanisms between them. Front-line healthcare workers fighting against COVID-19 are under high pressure due to both heavy workload and urgent time requirements. Research has shown that time pressure has a negative impact, and that individual depression is heavily influenced by the time urgency perceived [17]. In the stressor–outcome literature, scholars have suggested that anxiety, restlessness, and other stressors trigger an individual’s stress response, such as negative emotions and job burnout [18]. Scholars have noted the importance of time pressure and its relation to time constraints and individual perception of stress [19]. Healthcare workers who take responsibility for assisting or treating patients with COVID-19 are likely to experience heavy stress. Under the demands of time pressure, anxiety or restlessness may be aggravated, resulting in emotional exhaustion. Given the importance of understanding the impact of time pressure on the emotional exhaustion of front-line healthcare workers during the COVID-19 pandemic, our study fills a critical gap in the literature by explaining how time pressure influences emotional exhaustion in this context.

Second, we sought to uncover whether social sharing has a positive or negative impact on emotional exhaustion. The conservation of resources theory suggests that individuals experiencing a high level of pressure tend to use coping strategies to deal with negative emotions for the sake of conserving their psychological resources [14]. One of the most common ways that individuals engage in coping is by seeking social support, which is the assistance received from and/or concern perceived by others. When facing time pressures, healthcare workers may wish to relieve their stress and gain social support through sharing negative experiences with others, but the extended review and rumination of negative events may prolong their negative emotions, and thus consume their emotional resources. Hence, we predicted that social sharing would worsen emotional exhaustion rather than relieve it.

Third, we aimed to identify the moderating role of cognitive reappraisal in the relationship between time pressure and emotional exhaustion through social sharing. Healthcare workers undertake heavy burdens resulting from constrained time combined with arduous tasks and may vent their negative emotions through social sharing, such as complaining to others. However, if engaging in cognitive reappraisal, front-line healthcare workers may appraise their pressure in a positive way. They may regard their rescue burden as a glory mission due to their responsibility for saving lives. Thus, we predicted that people would decrease their social sharing (e.g., through complaining or sharing the negative event with others) when their cognitive reappraisal is high. Furthermore, increasing cognitive reappraisal is beneficial to reduce negative emotions and emotional exhaustion [20]. In contrast, we predicted that if individuals are low in cognitive reappraisal, they will have a greater emotional response to stressors and engage in more social sharing behaviors. In this way, time pressure may indirectly lead to increases in emotional exhaustion through social sharing.

## Methods

### Study design and data collection

This cross-sectional study was conducted in Wuhan, China between March 4 and March 19, 2020. To facilitate the implementation of the investigation, cluster random sampling methods were used to choose the participants.

We selected out three main kinds of hospitals from healthcare workers’ workplaces: infectious diseases specialist hospitals, general hospitals, and mobile medical hospitals. Among these three kinds of workplaces, the four selected were Huoshenshan hospital, Leishenshan hospital, Wuhan Xiehe hospital, and a mobile cabin hospital. The corresponding numbers of participants recruited from each hospital were 45, 45, 90, and 90, respectively.

We focused on front-line medical workers supporting Wuhan from Chongqing. Inclusion criteria were the following: 1) those who worked as frontline health workers and participated in the fight against COVID-19 in Hubei; 2) age ≥ 18 years; 3) able to read and complete the questionnaires independently; and 4) agreeable to participate voluntarily in this study. Exclusion criteria included people who were recently affected by major events other than COVID-19 or those with a history of neurasthenia and trauma.

Before the primary study, the questionnaire was piloted with a small number of participants to confirm the accuracy of the expression of content.

Taking the epidemic risk into account, the primary study was administered online to avoid unnecessary human contact. In total, 270 medical personnel based across three clusters were invited to participate in our investigation, and 232 participants completed the survey resulting in an effective response rate of 85.9%. As there were no previous studies in similar populations available, no formal sample size calculation was performed.

This study was approved by the Ethics Committee of Chongqing Maternal and Child Health Hospital. Informed consent from all individual participants was obtained prior to taking part in the research.

### Research instruments

The scales used are widely accepted in academic research. We followed the standard translation and back-translation procedures to ensure all the survey materials were accurately translated from English to Chinese. All the variables were scored on a five-point Likert scale ranging from 1 (strongly disagree) to 5 (strongly agree). Variables and measurement tools are as follows:

Time pressure was measured using a four-item scale [19]. In this study, the scale’s alpha coefficient for internal consistency was 0.821. The scale included items such as “In order to complete the task on time, we often have to bear a lot of pressure” and “The time used to complete the work is extremely constrained”.

Social sharing was measured by a four-item scale [12]. In this study, the scale’s alpha coefficient for internal consistency was 0.787. The scale included items such as “In the past period, I have talked with friends about unpleasant things that have happened at work”.

Cognitive reappraisal was measured by a six-item scale [15]. In this study, the scale’s alpha coefficient for internal consistency was 0.712. The scale included items such as “When I want to feel positive emotions (such as happiness), I change the way I think” and “When I’m under pressure, I think about things in a way that helps me stay calm”.

Emotional exhaustion was measured by a five-item scale [21]. In this study, the scale’s alpha coefficient for internal consistency was 0.876. The scale included items such as “I feel exhausted from work” and “I’m close to having a burnout because of work”.

Based on previous research and the current conditions of rescue work, we considered gender, age, educational background, and rescue experience to be control variables.

### Data analysis

AMOS 24.0 was used for confirmatory factor analysis (CFA) of the questionnaire, SPSS 25.0 for hierarchical regression analysis, independent-samples T-test and one-way ANOVA analysis, and PROCESS 3.3 for further testing such as the Johnson-Neyman technique and conditional process analysis. First, we standardized the independent variables, mediating variables, and moderating variables to avoid multicollinearity caused by the addition of interactions. Time pressure was used as a predictive variable, social sharing as a mediating variable, cognitive reappraisal as a moderating variable, and emotional exhaustion as an outcome variable.

### Quality control

All data were self-reported and were collected within a specific period of time, which may cause common method variance (CMV) bias. Hence, we used the single-factor test [22] to measure the degree of bias in the current study. According to the SPSS results, four factors were generated without rotation, explaining 61.276% of the variation. The first principal component obtained was 38.330%, indicating that all items reached an explained variance of 38.330% and did not exceed the recommended value of 50% [22]. Thus, the data quality of this study was deemed reliable and without unacceptable CMV bias.

## Results

### Demographic characteristics

The demographic characteristics of the participants are shown in Table 1. Across the 232 samples, 22.4% of the healthcare workers were male and 77.6% female. The average age of the participants was 33.88 years (SD = 0.71), ranging from 20 to 55 years. A total of 70.7% of healthcare workers reported that their highest level of education was university graduation; 15.90% had a college or lower degree; and 13.4% had completed a master’s degree or higher. Only 13.8% of the healthcare workers had rescue experience during a serious emergency before COVID-19.

**Table 1:** Demographic characteristics (*N* = 232)

Variable	Number	Percent
**Gender**		
Male	52	22.40%
Female	180	77.60%
**Age in years**		
< 20	0	0.00%
20–29	69	29.70%
30–39	124	53.40%
40–49	35	15.10%
> 50	4	1.70%
**Education**		
College or lower	37	15.90%
Undergraduate	164	70.70%
Postgraduate or above	31	13.40%
**Rescue experience**		
Yes	32	13.80%
No	200	86.20%
**Major**		
Clinical medicine	44	19.00%
Nursing	169	72.80%
Pharmacy, Radiology, Testing, Imaging	9	3.90%
Administrative management	6	2.60%
Others	4	1.70%

Table 2 reported the independent-samples T-test and one-way ANOVA analysis of emotional exhaustion in relation to demographic characteristics. Mean emotional exhaustion differed across the groups of gender, age, education, rescue experience, and major.

**Table 2:** EE and demographic characteristics in categorical items (*N* = 232)

Variable	Emotional Exhaustion	
	Mean	SD
**Gender**	*p* = 0.047	
Male	2.56	0.93
Female	2.27	0.75
**Age in years**	*p* = 0.002	
< 20	0	0
20–29	2.04	0.56
30–39	2.42	0.83
40–49	2.57	0.96
> 50	2.75	0.82
**Education**	*p* = 0.030	
College or lower	2.17	0.82
Undergraduate	2.31	0.77
Postgraduate or above	2.66	0.90
**Rescue experience**	*p* = 0.011	
Yes	2.67	0.98
No	2.28	0.76
**Major**	*p* = 0.014	
Clinical medicine	2.47	0.87
Nursing	2.29	0.76
Pharmacy, Radiology, Testing, Imaging	2.02	0.60
Administrative management	3.27	0.94
Others	1.90	1.09

Note:EE is emotional exhaustion

### Reliability and validity

The internal consistency for time pressure, social sharing, cognitive reappraisal, and emotional exhaustion was estimated using Cronbach’s coefficient alpha. These scores were all over 0.7, indicating satisfactory accuracy and reliability.

Given the data were obtained from a single source, we conducted CFA to evaluate the discriminant validity between variables. The four-factor model shown in Table 3 (χ^2^/df = 2.23, RMSEA = 0.07, IFI = 0.90, CFI = 0.90, SRMR = 0. 07) had a better fit to the data than the other models, and identified the independence between the four variables. Thus, we concluded that our variables had reasonable validity, and our data were not affected by homologous bias.

**Table 3:** Confirmatory factor analysis (*N* = 232)

Model	Factor	χ^**2**^	df	χ^**2**^/df	Δχ^**2**^	IFI	CFI	RMSEA	SRMR
1	Four-factor	287.26	129	2.23		0.90	0.90	0.07	0.07
2	Three-factor	481.28	132	3.65	194.02	0.79	0.78	0.11	0.10
3	Two-factor	669.26	134	4.99	382.00	0.67	0.67	0.13	0.12
4	One-factor	767.14	135	3.68	479.89	0.61	0.61	0.14	0.12

Note: Four-factor model: time pressure, social sharing, cognitive reappraisal, emotional exhaustion. Three-factor model: time pressure + cognitive reappraisal, social sharing, emotional exhaustion. Two-factor model: time pressure + cognitive reappraisal + social sharing, emotional exhaustion. One-factor model: time pressure + cognitive reappraisal + social sharing + emotional exhaustion

*IFI* incremental fit index, *CFI* comparative fit index, *RMSEA* root mean square error of approximation, *SRMR* standardized root mean square residual

### Descriptive statistics and correlations

Table 4 reports the descriptive statistics and correlations among the variables. Time pressure was positively related with social sharing (*r* = 0.22, *p* < 0.01) and emotional exhaustion (*r* = 0.56, *p* < 0.01). Social sharing was positively associated with emotional exhaustion (*r* = 0.16, *p* < 0.05), while time pressure was negatively related to cognitive reappraisal (*r* = 0.16, *p* < 0.05).

**Table 4:** Descriptive statistics and correlations (*N* = 232)

Variables	Mean	SD	1	2	3	4	5	6	7	8
1. Gender	0.22	0.42	1							
2. Age	33.88	0.71	0.25**	1						
3. Education	1.97	0.54	0.18**	0.37**	1					
4. Rescue Experience	0.14	0.35	0.3**	0.43**	0.16*	1				
5. Time Pressure	2.49	0.88	0.13*	0.21**	0.15*	0.1	1			
6. Social Sharing	2.31	0.92	−0.09	0.16*	0.19**	0.04	0.22**	1		
7. Cognitive Reappraisal	3.72	0.53	−0.04	−0.07	−0.07	−0.07	− 0.16*	− 0.17**	1	
8. Emotional Exhaustion	2.34	0.80	0.15*	0.24**	0.16*	0.17*	0.56**	0.25**	−0.25	1

Note: *p < 0.05, **p < 0.01 (two-tailed)

### Mediating role of social sharing in the relationship between time pressure and emotional exhaustion

We used hierarchical linear regression to test the mediating effect. As shown in Table 5, time pressure was positively associated with social sharing (*β* = 0.199, *p* < 0.05) and emotional exhaustion (*β* = 0.527, *p* < 0.001). Social sharing was also positively related with emotional exhaustion (*β* = 0.232, *p* < 0.001).

**Table 5:** Indirect effect of social sharing (*N* = 232)

Variable	Social Sharing			Emotional Exhaustion		
	Step 1(β)	Step 2(β)	Step 1(β)	Step 2(β)	Step 3(β)	Step 4(β)
Gender	−0.155*	−0.170*	0.076	0.035	0.112*	0.058
Age	0.134	0.102	0.167*	0.082	0.136	0.069
Education	0.163*	0.149*	0.078	0.039	0.04	0.02
Rescue Experience	0.001	0.001	0.059	0.06	0.059	0.06
Time Pressure		0.199*		0.527***		0.501***
Social Sharing					0.232***	0.132*
R^2^	0.066	0.103	0.074	0.336	0.124	0.352
F	4.012**	3.208***	4.542**	22.916***	6.426***	20.366***
ΔR^2^	0.066	0.037	0.074	0.262	0.05	0.016
ΔF	4.012**	9.396**	4.542**	89.341***	12.998***	3.389*

Note: *p < 0.05, **p < 0.01, ***p < 0.001 (two-tailed)

Social sharing had a partial mediating effect in the relationship between time pressure and emotional exhaustion as the regression coefficient for time pressure decreased when social sharing was added (from *β* = 0.527 to *β* = 0.501, *p* < 0.001).

To verify the mediating effect further, we carried out 5000 bootstraps for deviation correction. The results reported that the indirect effect of social sharing was 0.026, and the confidence interval (CI) was 0.003, 0.061, which did not include the value of 0. Thus, the result confirmed the significant mediating role of social sharing in the relationship between time pressure and emotional exhaustion. In addition, the direct effect was also significant (CI = 0.376, 0.621), which indicated that social sharing had a partially mediating effect.

### Moderating influence of cognitive reappraisal

We used hierarchical linear regression to test the moderating effect. In step 1, we entered all control variables into the regression. In step 2, we entered time pressure and cognitive reappraisal as predictors. In step 3, we entered the interaction term, which was significantly related to social sharing (*β* = − 0.137, *p* < 0.05). Cognitive reappraisal proved to negatively moderate the relationship between time pressure and social sharing (*β* = − 0.137, *p* < 0.05).

Additionally, we calculated the simple slope of the moderating effect. We divided cognitive reappraisal into the different levels of low, moderate, and high by calculating the value of the standard deviation minus one, the standard deviation, and the standard deviation plus one, respectively. The results demonstrated that a low level of cognitive reappraisal was significantly related to social sharing (E = 0.319, *t* = 3.492, *p* < 0.001).

**Fig. 2:**
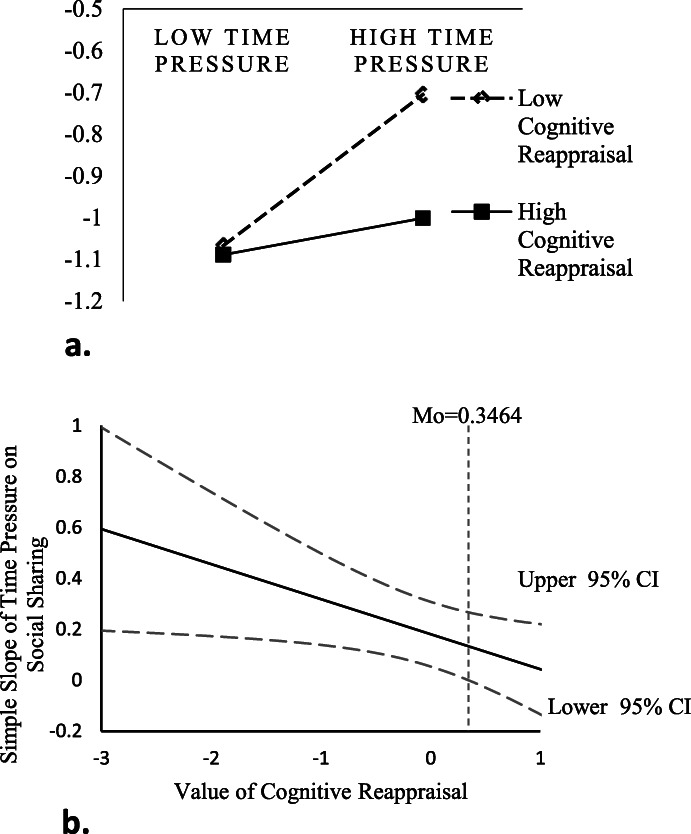
**a** Moderate Influence of Cognitive Reappraisal (*N* = 232). **b**. Johnson-Neyman Plot of the Interaction between Time Pressure and Cognitive Reappraisal (*N *= 232)

According to Fig. 2a and the results above, cognitive reappraisal interacted with time pressure such that the positive relationship between time pressure and social sharing became stronger when cognitive reappraisal was lower.

Furthermore, we conducted Johnson-Neyman technique analysis to identify quantitative change along the continuum of the moderator cognitive reappraisal between a statistically significant and nonsignificant effect of time pressure. As shown in Fig. 2b, at a 95% confidence level, the effect of time pressure on social sharing was significant when cognitive reappraisal ≤0.3464.

### Conditional process analysis

Based on the results above, we further tested the moderated mediation model using conditional process analysis. For three different levels of cognitive reappraisal, we calculated the different significance of the indirect effect of time pressure on emotional exhaustion through social sharing. As shown in Table 6, specifically, the indirect effect of time pressure on emotional exhaustion was stronger when cognitive reappraisal was lower (Effect = 0.042, CI = 0.005, 0.097) rather than higher (Effect = 0.006, CI = -0.023, 0.032). Therefore, cognitive reappraisal was found to moderate the indirect effect of time pressure on emotional exhaustion through social sharing.

**Table 6:** Conditional process analysis

Cognitive Reappraisal	Effect	BootSE	BootLLCI	BootULCI
Low Level	0.042	0.024	0.005	0.097
Moderate Level	0.024	0.014	0.002	0.056
High Level	0.006	0.013	−0.023	0.032

Note: Bootstrap is set by a 95% confidence interval for 5000 repeated samples

*BootSE* bootstrap standard error, *BootLLCI* lower limit of the bootstrap confidence interval, *BootULCI* upper limit of the bootstrap confidence interval

## Discussion

We recruited front-line healthcare workers during the COVID-19 pandemic to examine how time pressure can lead to emotional exhaustion. The results showed that time pressure was positively related to emotional exhaustion, and that social sharing played a positive mediating role. Moreover, cognitive reappraisal buffered the effect of time pressure on social sharing, and the level of emotional exhaustion became relatively low after the addition of cognitive reappraisal.

We found that the emotional exhaustion of healthcare workers was positively related to the time pressure they perceived. Previous studies have suggested that time pressure may result in both positive and negative consequences [23, 24]. Our research demonstrated that time pressure was more likely to bring a negative effect on healthcare workers than a positive effect during the COVID-19 epidemic. In terms of healthcare workers during this time, the relationship between time pressure and emotional exhaustion has remained largely unexplored, and our findings provide new insight into the processes involved.

The emotional exhaustion of front-line healthcare workers was affected not only by direct stressors but complicated psychological processes. Previous scholars have shown a positive effect of social sharing, but little research has considered the potentially negative influence. Although social sharing is commonly regarded as a positive strategy for coping with pressure, in our study it was found to heighten emotional exhaustion in the COVID-19 period. Our results are consistent with the perspective of Baranik [12], who argues that although people might gain social support (such as encouragement) through social sharing, the extended consideration of negative events may instead prolong their negative experience, adversely affecting emotion.

We found that cognitive reappraisal interacted with time pressure such that the positive relationship between time pressure and social sharing was stronger when cognitive reappraisal was lower. Furthermore, cognitive reappraisal moderated the indirect effect of time pressure on emotional exhaustion through social sharing. Cognitive reappraisal was considered in the literature to be the most effective way of regulating emotions. Previous studies have suggested that cognitive reappraisal can mitigate the effect of negative events and reduce individuals’ negative experiences [20, 25]. The negative moderating role of cognitive reappraisal in our study is of theoretical importance to understanding healthcare workers’ emotional regulation during the COVID-19 epidemic. Beyond this, our findings have important implications for hospital administrators when organizing medical teams in the event of such infectious emergencies.

It is important to understand the mental health response of healthcare workers after a public health emergency. It is also important to formulate recruitment evaluation criteria of factors, such as psychological state, stress coping, and emotion regulation ability, to allow the selection of healthcare workers with outstanding emotional adjustment ability who will be able to cope with pressure generally and also during times of crisis. In addition, team leaders and administrators who can positively guide healthcare workers to regulate negative emotions should be considered preferentially.

Positive emotions help prevent work-related stress and exhaustion. Research has identified the important effect of mindfulness-based stress reduction on psychological symptoms [26]. We suggest that front-line healthcare workers choose sensible working shifts, promote self-management, and carry out pressure relief activities based on mindfulness to reduce the risk of exhaustion. For team leaders and administrators, it would be conducive to organize mindfulness-based and self-management activities to improve team members’ abilities to engage in cognitive reappraisal and thus prevent potential burnout.

As for hospitals, it is advisable to provide front-line healthcare workers with a comfortable work environment to help relieve pressure in the workplace. For example, managers should improve workflow to ensure that healthcare workers have sufficient rest opportunities and exercise time. Hospital administrators can also ensure the provision of key medical supplies and daily necessities to help healthcare workers feel valued, thus helping prevent exhaustion or burnout.

### Limitations and directions for future research

Although the present study has several strengths, the limitations need to be considered. The limitations are twofold. First, this study was a cross-sectional design and all data were self-reported, which may introduce common method biases. Although our results were found to be reliable, future research should collect multisource, multilevel data to achieve more accurate outcomes, and use the longitudinal method to capture dynamic fluctuations as well as explain causality between variables.

Second, only Chinese healthcare workers were recruited, which may limit the generalizability of our current conclusions. Because of traditional collectivism values, the Chinese population may tend to adopt more social sharing behaviors when dealing with pressure than those with individualistic values. Future research could replicate and extend our findings by collecting samples from different countries.

## Conclusions

Understanding emotional exhaustion in front-line healthcare workers is a significant issue for public health and emergency management. We identified that although social sharing is commonly regarded as a positive behavior, there may be a dark side in terms of its impact on healthcare workers during the COVID-19 pandemic. Improving cognitive reappraisal may present a positive strategy toward alleviating emotional exhaustion.

## Data Availability

The datasets used and analysed during the current study are available from the corresponding author on reasonable request.
